# Is water exchange superior to water immersion in detecting adenomas during colonoscopies? Results from a Bayesian network meta-analysis

**DOI:** 10.18632/oncotarget.25504

**Published:** 2018-07-17

**Authors:** Xin Shi, Dan Tian, Xiaofei Ye, Qiong Wu, Yanglin Pan, Zhiping Yang, Daiming Fan

**Affiliations:** ^1^ State Key Laboratory of Cancer Biology, National Clinical Research Center for Digestive Diseases and Xijing Hospital of Digestive Diseases, Fourth Military Medical University, Xi’an, China; ^2^ Office of Educational Administration, Fourth Military Medical University, Xi’an, China; ^3^ Department of Health Statistics, Second Military Medical University, Shanghai, China

**Keywords:** colonoscopy, water exchange, water immersion, colorectal adenoma, network meta-analysis

## Abstract

**Aim:**

Water-assisted colonoscopy (water exchange [WE] and water immersion [WI]) has been shown to improve the adenoma detection rate. However, few studies have compared these two methods head-to-head. Thus, we conducted a network meta-analysis to integrate both direct and indirect evidence comparing the effectiveness of these two procedures.

**Method:**

We searched PubMed, Web of Science, and the Cochrane Central Register of Controlled Trials for original papers and abstracts published up to March 2018. Randomized controlled trials (RCTs) reporting data in accordance with the eligibility criteria were included in this study. We performed a Bayesian random effects network meta-analysis with mixed comparisons.

**Results:**

Twenty-nine studies (*n* = 11464 patients) including 6 direct and 23 indirect comparisons were included in this network meta-analysis. There was a statistically significant difference in the efficacy of adenoma detection when WE was compared with WI (risk ratio [RR]: 1.2, 95% credible interval [CrI]: 1.1–1.3), air insufflation (AI; RR: 1.3, 95% CrI: 1.1–1.4), and carbon dioxide (CO_2_) insufflation (RR: 1.2, 95% CrI: 1.1–1.5). The different methods were ranked in order from the most to least effective in adenoma detection as follows: WE, WI, AI, and CO_2_. Moreover, although there were no significant differences in pain scores, willingness to repeat, caecal intubation rate, or total procedure time between WI and WE colonoscopy, WE required a longer caecal intubation time than WI.

**Conclusion:**

This network meta-analysis supposes that WE may be superior to WI in detecting adenomas during colonoscopies without affecting other technical features or patient acceptance.

## INTRODUCTION

A colonoscopy is considered the gold standard for diagnosing colonic diseases and an important therapeutic modality [1]. It can reduce the risk of death from colorectal cancer by detecting tumours at an earlier, more treatable stage and removing precancerous adenomas [2, 3]. The adenoma detection rate (ADR), defined as the proportion of patients with at least one adenoma detected during a screening colonoscopy in average-risk patients >50 years, has been shown to be inversely associated with the development of interval colorectal cancer [4, 5]. In our previous study, the recurrence rates of adenomas among post-polypectomy patients were high [6]. Thus, it is essential to improve the ADR to benefit patients undergoing a colonoscopy [7, 8].

Traditionally, a diagnostic colonoscopy begins with air insufflation (AI) to distend the colonic lumen to permit visualization and passage. Gas insufflation with either air or carbon dioxide (CO_2_) can be painful and poorly tolerated by patients [9, 10]. Due to these undesirable outcomes, investigators adopted the use of water-aided methods in lieu of gas insufflation to improve comfort during the insertion procedure. Water-aided methods comprise two major categories, namely, water immersion (WI) and water exchange (WE). WI is characterized by suction removal of the infused water during the withdrawal phase of a colonoscopy, and WE is characterized by suction removal of the infused water predominantly during the insertion phase [11, 12].

Recent studies revealed that, compared with AI, WE significantly improved the ADR [13, 14]. However, previous systematic reviews and meta-analyses showed controversial results regarding the differences between the ADRs of water infusion (water immersion or water exchange methods) and standard air insufflation for colonoscopies [15–20] (Supplementary Table 1). Hafner S [15] found that adenoma detection rate was slightly improved with water infusion (risk ratio 1.16, 95% CI 1.04–1.30, *P* = 0.007). But others showed no significant statistical differences. To our knowledge, there are few studies with head-to-head comparisons of the ADRs associated with WE and WI. Thus, we conducted a network meta-analysis to determine the comparative effectiveness of different water-assisted methods during colonoscopy.

## METHODS

The methodology and reporting of this study complies with the PRISMA statement for reporting systematic reviews that incorporated network meta-analyses of health care interventions [21].

### Eligibility criteria

For this network meta-analysis, we only considered randomized controlled trials (RCTs) irrespective of publication status or date of publication. We excluded studies of other design because of the risk of bias associated with such studies. We included RCTs with participants who underwent a colonoscopy with or without sedation. We included RCTs that compared WI or WE with AI or CO_2_, and we excluded other assisted colonoscopy methods such as cap-assisted WI, water-assisted plus indigo carmine, and water-assisted plus CO_2_. Studies reporting both included and excluded populations were considered only if subject data specifically meeting the inclusion criteria for this network meta-analysis could be extracted (e.g., data for subjects who received WE as a single method in a trial comparing WE alone vs. WE with additional CO_2_). Abstracts reporting unpublished studies were also considered if sufficient data were reported.

### Information sources and search

A bibliographic search was performed using PubMed, Web of Science, and the Cochrane Central Register of Controlled Trials to identify original studies analysing the efficacy of water-assisted colonoscopy. The search strategy was as follows: colonoscopy, water and randomized or randomized trial or clinical trial. Moreover, reference lists from the retrieved articles, systematic reviews and meta-analyses were evaluated to identify additional relevant studies. To obtain the maximum number of papers, no publication date restrictions were imposed in any database. The last search was completed in March 2018. Only English language publications were included in this study.

### Study selection

Two investigators (X.S. and D.T.) independently identified trials for inclusion by screening titles and abstracts yielded by the search. To potentially include additional studies, we searched for full-length articles of all references from the studies that the investigators had identified in the initial search. We selected trials for inclusion based on the review of these full-length articles. Queries concerning inclusion were resolved by discussion and consensus between the two investigators.

### Data extraction

Two investigators (X.S. and D.T.) independently extracted the following data from each study and summarized the data in tables: first author’s name, publication year, country, number of centres, registration numbers, interventions, indications, sedation, sample size, percentage of males, age of each intervention, number of participants per group, number of participants per group with at least one detected adenoma, pain score, willingness to repeat colonoscopy, number of participants per group who completed caecal intubation, caecal intubation time, and total procedure time. Differences in opinion between the investigators were resolved through discussion. Pain scores were derived from 0 to 10 visual analogue or numeric rating scales with 0 = none and 10 = most severe pain; if an included study reported 0–5 or other visual analogue scales, then the scores were converted. For continuous data, if an included RCT with a sample size larger than 100 reported only the median and interquartile range (IQR), then the median was regarded as the mean, and the standard deviation was calculated as IQR/1.35 [22].

### Risk of bias assessment

We assessed the risk of bias as described in the Cochrane Handbook for Systematic Reviews of Interventions [23]. Bias risk assessment was performed with reference to the following domains: random sequence generation (selection bias), allocation concealment (selection bias), blinding of participants and personnel (performance bias), blinding of outcome assessment (detection bias), incomplete outcome data (attrition bias), selective reporting (reporting bias) and other detectable biases. Each risk of bias component was rated as low risk, unclear risk, or high risk of bias. A study with sufficient data to fulfill the criteria for the quality item was marked “low risk of bias”. A study in which the reported data did not fulfill the criteria for the quality item was marked “high risk of bias”. “Unclear risk of bias” was marked for studies that did not report the necessary data to assess the quality item.

### Outcome measures

The primary outcome measure was the ADR, which was defined as the proportion of patients in whom at least one adenoma was detected during a colonoscopy. Secondary outcome measures were the mean and maximum pain scores (defined as the mean and maximum pain scores of patients during the colonoscopy), willingness to repeat rate (defined as the proportion of patients who were willing to repeat the procedure after the colonoscopy), caecal intubation rate, caecal intubation time, and total procedure time.

### Statistical analysis

Given the limited number of studies that directly compared WE and WI, we used a network meta-analytical approach to simultaneously combine direct comparisons of these interventions within studies and, if available, indirect comparisons between studies. Whenever possible, we used results from intention-to-treat analyses. Network meta-analysis was conducted by using a Bayesian Markov-chain Monte Carlo method, and data were fitted in R statistical software version 3.3.2 (R Foundation for Statistical Computing, Vienna, Austria). Analytical results were presented as risk ratios (RRs) and mean differences (MDs) with 95% credible intervals (CrIs). A network diagram was used for each outcome to present the comparisons between different water-assisted colonoscopy methods and common AI or CO_2_. In these diagrams, nodes represented different methods of colonoscopies, lines indicated direct comparisons and line thickness was proportional to the number of available studies. We ranked methods of colonoscopy based on analysis of ranking probabilities derived from Monte Carlo simulations [24]. The probabilities of different rankings of the same intervention were summed to 100%. Inconsistency between direct and indirect evidence was assessed by a “node-splitting” approach [25].

We did not perform any subgroup analysis or sensitivity analysis due to the lack of data. We conducted Egger’s regression asymmetry test to examine potential publication bias in terms of ADR by using STATA software version 12.0 (Stata Corporation, College Station, Texas, USA).

## RESULTS

### Study selection

**Figure 1:**
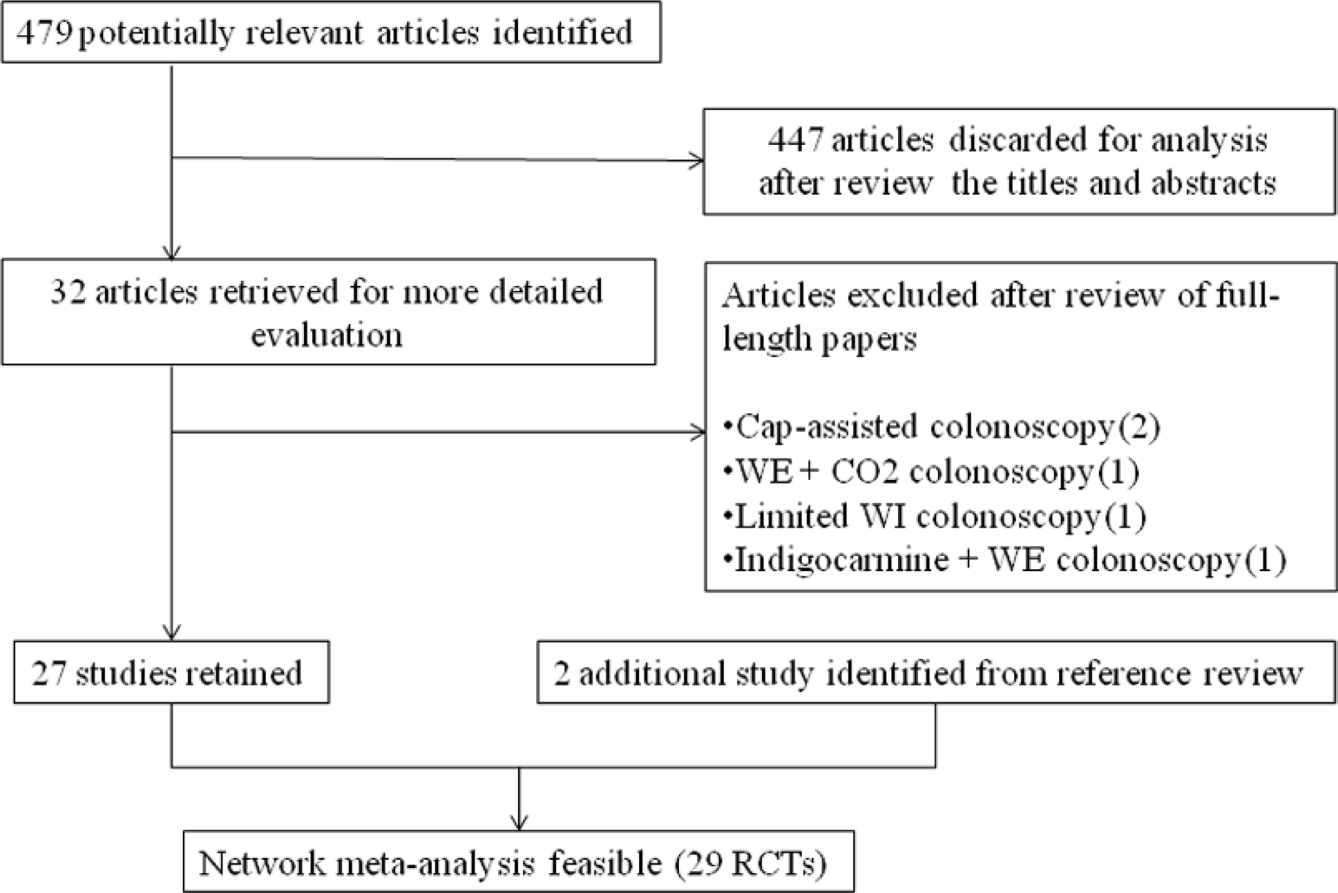
Study flowchart

Figure 1 shows the detailed steps of the study selection process. The literature search yielded 479 potentially relevant studies. Of these, 32 potentially eligible studies were retrieved from the electronic databases, and 2 additional relevant studies were identified through a manual search of the reference section of identified articles and former meta-analyses, yielding a total of 34 studies. After excluding 5 studies based on the predefined inclusion criteria, 29 studies were included in the network meta-analysis.

### Study characteristics and network geometry

Study characteristics and outcome measures of the 29 included studies are summarized in Table 1 and Supplementary Table 2. Six trials [26–31] including 2088 patients (18.2%) were analysed in per-protocol, others were analysed in intention-to-treat. The studies were published between 2009 [32] and 2017 [13, 14, 33]. Study population sizes ranged from 23 [34] to 3303 [13], and 11464 patients were included in total, which equated to 1998 patients in the WI group, 3778 patients in the WE group, 4431 patients in the AI group, and 696 patients in the CO_2_ group. The proportion of male patients ranged from 30.4% [34] to 100% [31], and the mean patient age varied from 51.1 [35] to 69 [34] years in the WI group, 36 [36] to 65.6 [27] years in the WE group, 40.2 [36] to 67 [34] years in the AI group, and 54.3 [26] to 63.1 [27] years in the CO_2_ group.

**Table 1:** Study characteristics

Author	Year	Country	Centres	Registration No.	Interventions	Indications	Sedation	Sample size	Male (%)	Age (y, mean ± SD)			
										WI	WE	AI	CO_2_
Cadoni S [14]	2017	Italy, Czech	3	NCT02041507	WI, WE, AI	Scr	On-demand	1224	54.9	61 ± 6.3	61.4 ± 6.2	60.9 ± 6.2	-
Jia H [13]	2017	China	6	NR	WE, AI	Scr, Sur, and Dx	Sedated	3303	51	-	50.3 ± 13.7	50.3 ± 13.9	-
Cadoni S [33]	2017	Italy, Czech	2	NCT02409979	WE, CO_2_	Dx	On-demand	240	61.7	-	59 ± 13.1	-	58 ± 13.7
Hsieh YH [37]	2017	China	2	NCT01894191	WI, WE, AI	Scr, Sur, and Dx	Sedated	651	51.6	55.9 ± 10.2	55.7 ± 10.6	54.8 ± 10.5	-
Arai M [27]	2016	Japan	2	UMIN000009706	WE, CO_2_	NR	Unsedated	403	61.3	-	65.6 ± 12	-	63.1 ± 12.6
Xu X [26]	2016	China	1	NR	WI, AI, CO_2_	Dx	Unsedated	287	48.3	54.3 ± 12.4	-	55 ± 10.7	54.3 ± 11.7
Falt P [36]	2015	Czech	1	NCT01933867	WE, AI	Dx	On-demand	92	53.3	-	36 ± 13	40.2 ± 14.6	-
Cadoni S [48]	2015	Italy	2	NCT01781650, NCT01780818	WI, WE, AI	Scr, and Dx	On-demand	576	58.7	60 ± 10.8	59 ± 11.3	59 ± 11.3	-
Cadoni S [49]	2015	Italy, Czech	2	NCT01954862	WI, WE, AI, CO_2_	Scr, and Dx	On-demand	624	58.7	59 ± 12.3	58 ± 15.5	58 ± 12.7	59 ± 12.2
Wang X [28]	2015	China	1	NCT01735266	WE, AI	94-95% Dx	Unsedated	300	47.3	-	46.6 ± 11.8	48.6 ± 13.2	-
Miroslav V [50]	2014	Yugoslavia	1	NR	WI, AI	Scr, Sur, and Dx	Unsedated	122	47.5	54.4 ± 14.8	-	58 ± 13	-
Hsieh YH [38]	2014	China	1	NCT01535326	WI, WE, AI	Scr, Sur, and Dx	Minimal	270	62.2	54.3 ± 11.4	56.9 ± 10.3	56.5 ± 13.7	-
Cadoni S [29]	2014	Italy	2	NCT01463319	WE, AI	Scr, Sur, and Dx	On-demand	672	60.3	-	58 ± 12.4	60 ± 12.3	-
Leung J [39]	2013	USA	1	NCT01383252	WE, AI	NR	Unsedated	100	97	-	61 ± 7	60 ± 6.7	-
Amato A [40]	2013	Italy	1	NCT01259583	WI, AI, CO_2_	Scr, Sur, and Dx	Unsedated	341	64.5	60 ± 11.5	-	60 ± 13.4	61.5 ± 14
Luo H [51]	2013	China	1	NCT01485133	WE, AI	Scr, Sur, and Dx	Unsedated	110	30.9	-	55.8 ± 11	56.6 ± 12	-
Bayupurnama P [35]	2013	Indonesia	1	NCT01341847	WI, AI	Dx	Unsedated	110	65.5	51.1 ± 14.7	-	50.4 ± 15.9	-
Hsieh YH [52]	2013	China	1	NCT0090555	WI, WE, AI	Scr, Sur, and Dx	Minimal	200	62	53 ± 12	57 ± 11	57 ± 13	-
Portocarrero DJ [34]	2012	USA	2	NR	WI, AI	Scr	Unsedated	23	30.4	69 ± 10	-	67 ± 15	-
Falt P [30]	2012	Czech	1	NCT01440543	WI, AI, CO_2_	Scr, Sur, and Dx	Minimal	420	51.7	60.1 ± 13.9	-	58.7 ± 13.8	59.4 ± 14.5
Hsieh YH [44]	2011	China	1	NR	WI, AI	Dx	Minimal	153	56.9	52.4 ± 13.5	-	56.3 ± 13.2	-
Ramirez FC [41]	2011	USA	1	NR	WE, AI	Scr	Minimal	368	96.5	-	60 ± 0.5	59.3 ± 0.5	-
Pohl J [42]	2011	Germany	1	DRKS00000431	WI, AI	Scr, Sur, and Dx	On-demand	116	73.3	62.7 ± 9.7	-	61.7 ± 11.5	-
Leung J [43]	2011	USA	1	NCT00920751	WI, AI	Scr, Sur	On-demand	100	99	60.7 ± 8.1	-	58.3 ± 7	-
Leung FW [47]	2010	USA	1	NCT00747084	WI, AI	Scr, Sur, and Dx	Unsedated	82	NA	66 ± 8.6	-	66.8 ± 8.4	-
Leung CW [31]	2010	USA	1	NCT00671177	WI, AI	Scr, Sur, and Dx	Minimal	229	100	62.5 ± 8.9	-	62.5 ± 8.9	-
Radaelli F [46]	2010	Italy	1	NCT00905554	WI, AI	Scr, Sur, and Dx	On-demand	230	58.3	58.4 ± 11.5	-	58.8 ± 13.3	-
Ransibrahmanakul K [45]	2010	USA	1	NCT00841282	WI, AI	Scr, Sur	Minimal	62	98.4	61 ± 7.9	-	61 ± 7.8	-
Leung JW [32]	2009	USA	1	NCT00785889	WI, AI	Scr, Sur	Minimal	56	91.1	60 ± 6.6	-	59 ± 8.6	-

USA, United States of America; NR, not reported; WI, water immersion; WE, water exchange; AI, air insufflation; CO_2_, carbon dioxide; Scr, screening; Sur, surveillance; Dx, diagnosis.

In 18 studies evaluating the ADR [13, 14, 27, 29–31, 34, 37–47], these rate ranged from 25% [46] to 54.5% [34] in the WI group, 18.3% [13] to 67.5% [27] in the WE group, 13.3% [13] to 46% [41] in the AI group, and 26.5% [30] to 57.8% [27] in the CO_2_ group. Of 27 studies evaluating pain scores [13, 26–40, 42–52], 13 studies evaluated the mean pain score [27, 29–32, 34–36, 40, 44, 46, 50, 52], which ranged from 1.3 [32] to 4.1 [31, 35, 50] in the WI group, 1.3 [29]to 4.3 [27] in the WE group, 2.3 [29, 30] to 6.4 [35] in the AI group, and 2 [30] to 4.8 [27] in the CO_2_ group, whereas 14 studies evaluated the maximum pain score [13, 26, 28, 33, 37–39, 42, 43, 45, 47–49, 51], which ranged from 2.3 [43] to 4 [49] in the WI group, 1.1 [28] to 3.3 [33] in the WE group, 2.9 [28] to 5.7 [26] in the AI group, and 2.9 [26] to 4.7 [33] in the CO_2_ group. In 21 studies evaluating the willingness to repeat rate [13, 14, 26, 28, 32, 34, 35, 38–40, 42–52], this rate ranged from 72.4% [42] to 100% [34, 38, 52] in the WI group, 76% [39] to 100% [38, 52] in the WE group, 48% [39] to 98.9% [38] in the AI group, and 88.7% [40] to 93.8% [26] in the CO_2_ group. In 27 studies evaluating caecal intubation time [13, 14, 26–33, 35–49, 51, 52], this timing ranged from 5.6 minutes [44] to 34 minutes [47] in the WI group, 6.9 minutes [41] to 17.5 minutes [52] in the WE group, 4.6 minutes [44] to 37 minutes [47] in the AI group, and 5 minutes [40] to 9.8 minutes [33] in the CO_2_ group. In 22 studies evaluating total procedure time [14, 26, 30–37, 39–49, 51], the total timing ranged from 14 minutes [34] to 56 minutes [47] in the WI group, 15.7 minutes [36] to 29 minutes [39] in the WE group, 13.1 minutes [44] to 56 minutes [47] in the AI group, and 13 minutes [40] to 20.5 minutes [49] in the CO_2_ group.

**Figure 2:**
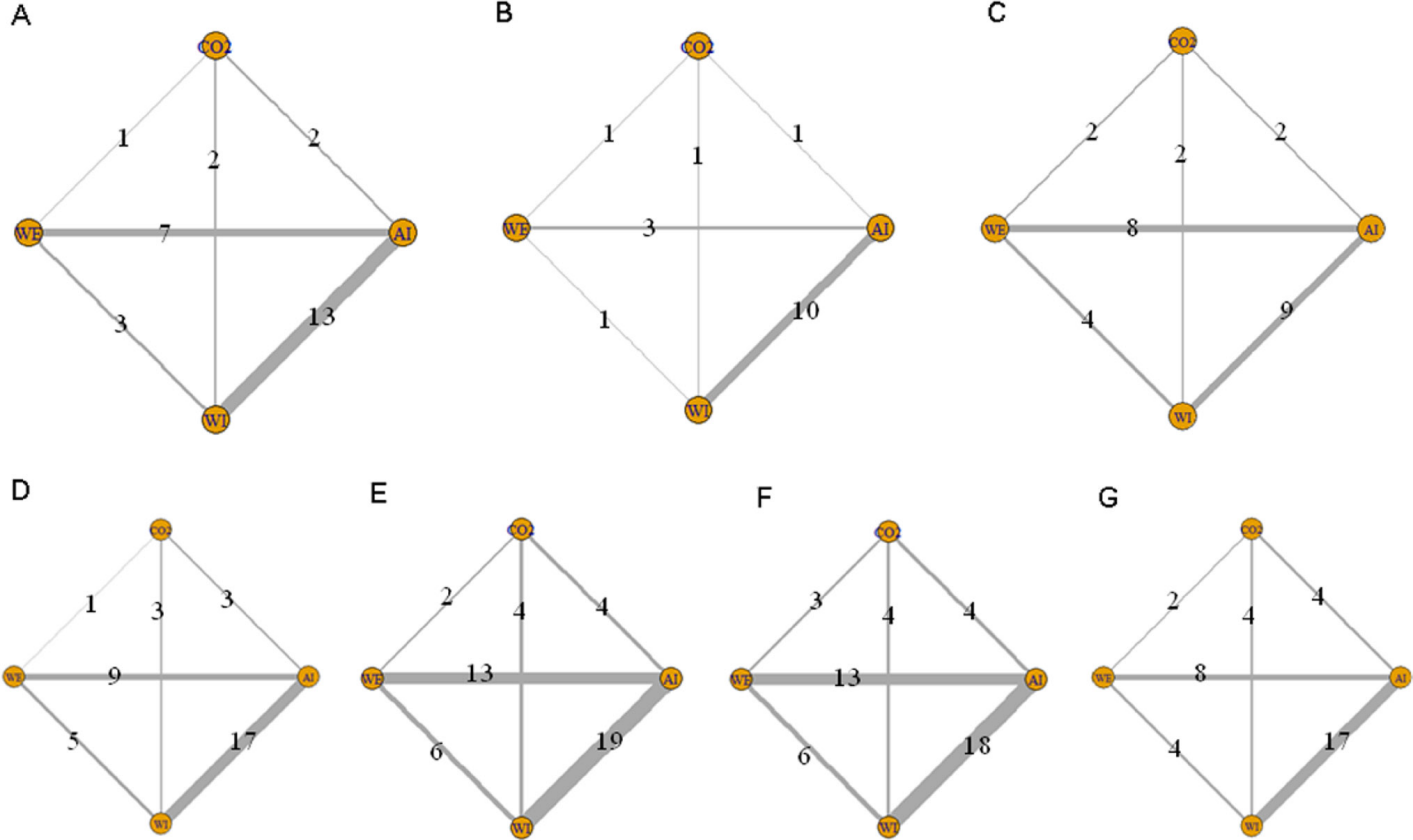
Network of comparisons included in the analyses Solid lines represent direct comparisons within the RCTs. Numbers denote trials that compared corresponding interventions. (**A**) ADR; (**B**) mean pain scores; (**C**) maximum pain scores; (**D**) willingness to repeat; (**E**) caecal intubation rate; (**F**) caecal intubation time; and (**G**) total procedure time.

Figure 2 shows the network of comparisons for each outcome of interest. In relation to ADR, 3 trials compared WI and WE, 7 trials compared WE and AI, 1 trial compared WE and CO_2_, 13 trials compared WI and AI, 2 trials compared WI and CO_2_, and 2 trials compared AI and CO_2_.

### Quality of trials

**Figure 3:**
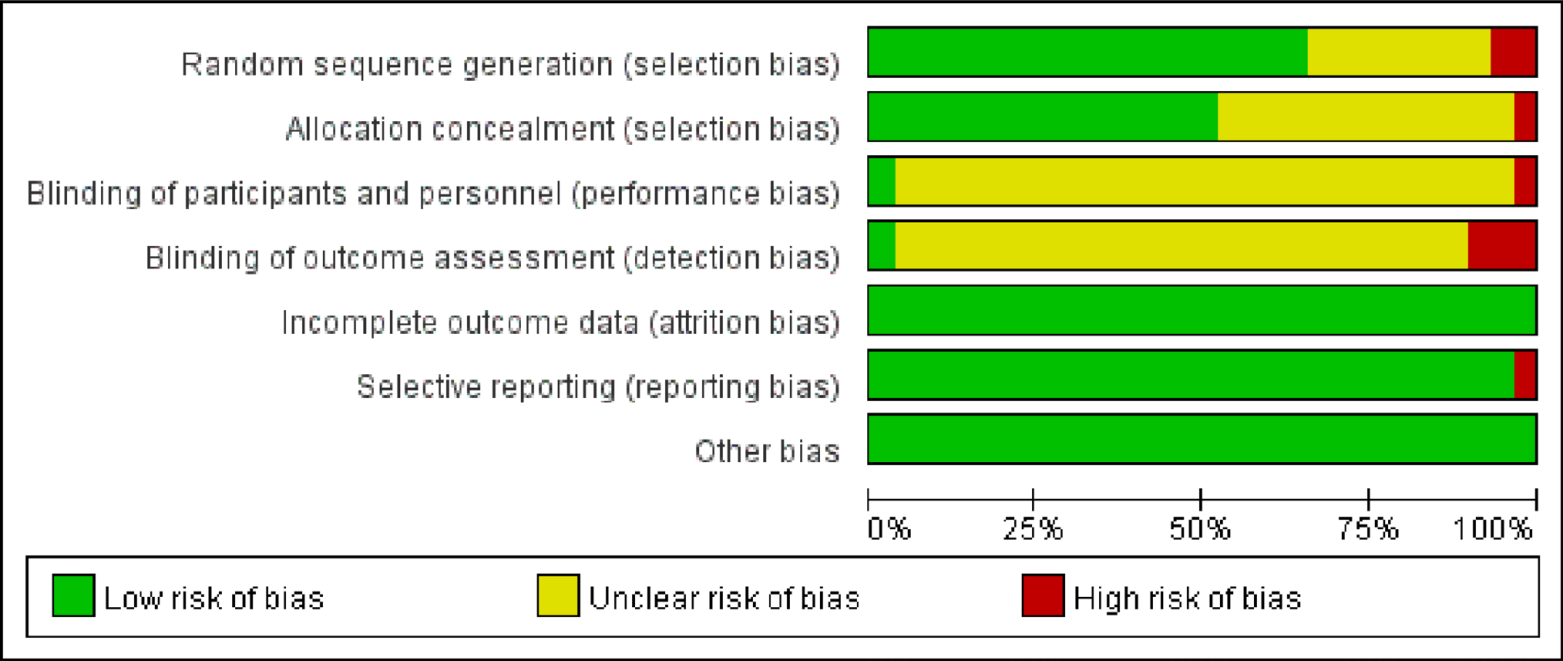
Risk of bias graph

The quality of the studies included in the network meta-analysis, as assessed using a risk of bias assessment tool, is shown in Figure 3 and Supplementary Figure 1. In relation to random sequence generation, most of the trials were rated as having a “low risk of bias” (19 of 29 trials), and regarding allocation concealment, 50% of trials were rated as having a “low risk of bias” (15 of 29 trials) because they used sealed opaque envelopes. Trials rated as having an “unclear risk of bias” had issues related to blinding of participants and personnel and of outcome assessment (27 and 26, respectively). In terms of attrition bias, reporting bias, and other biases, all trials were rated as having a “low risk of bias” except one [29], which was rated as having a “high risk of bias” in relation to reporting bias.

### Meta-analysis

**Figure 4:**
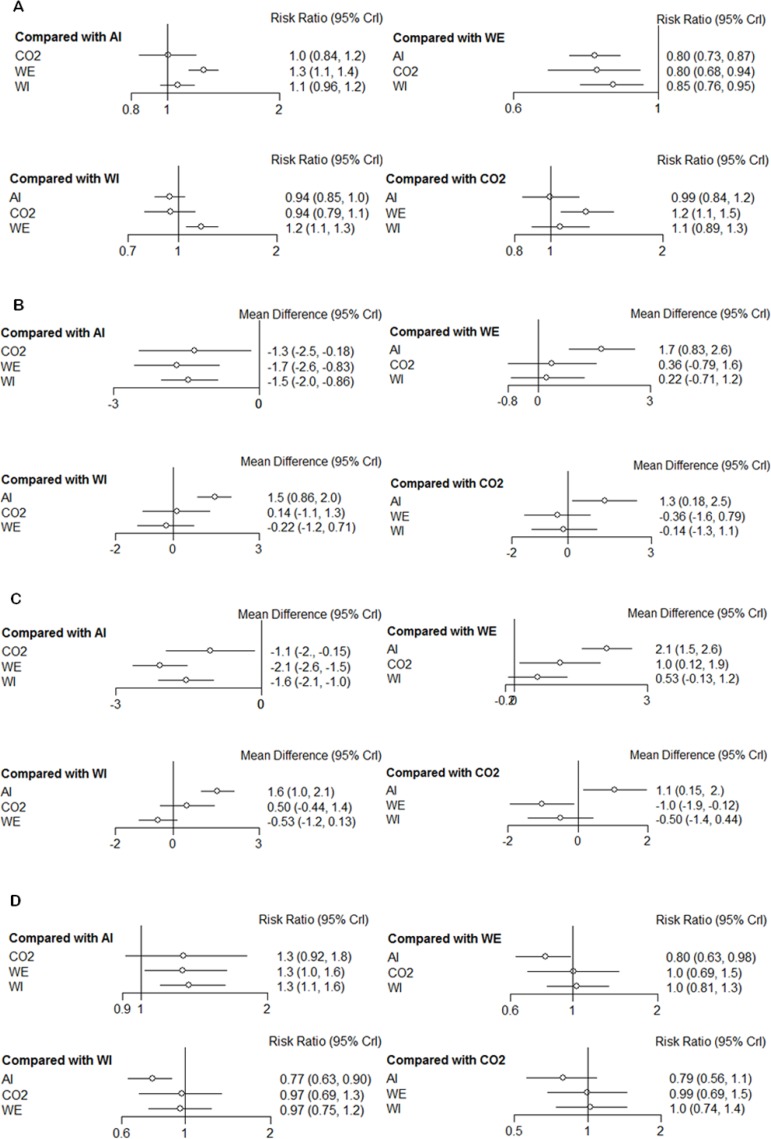

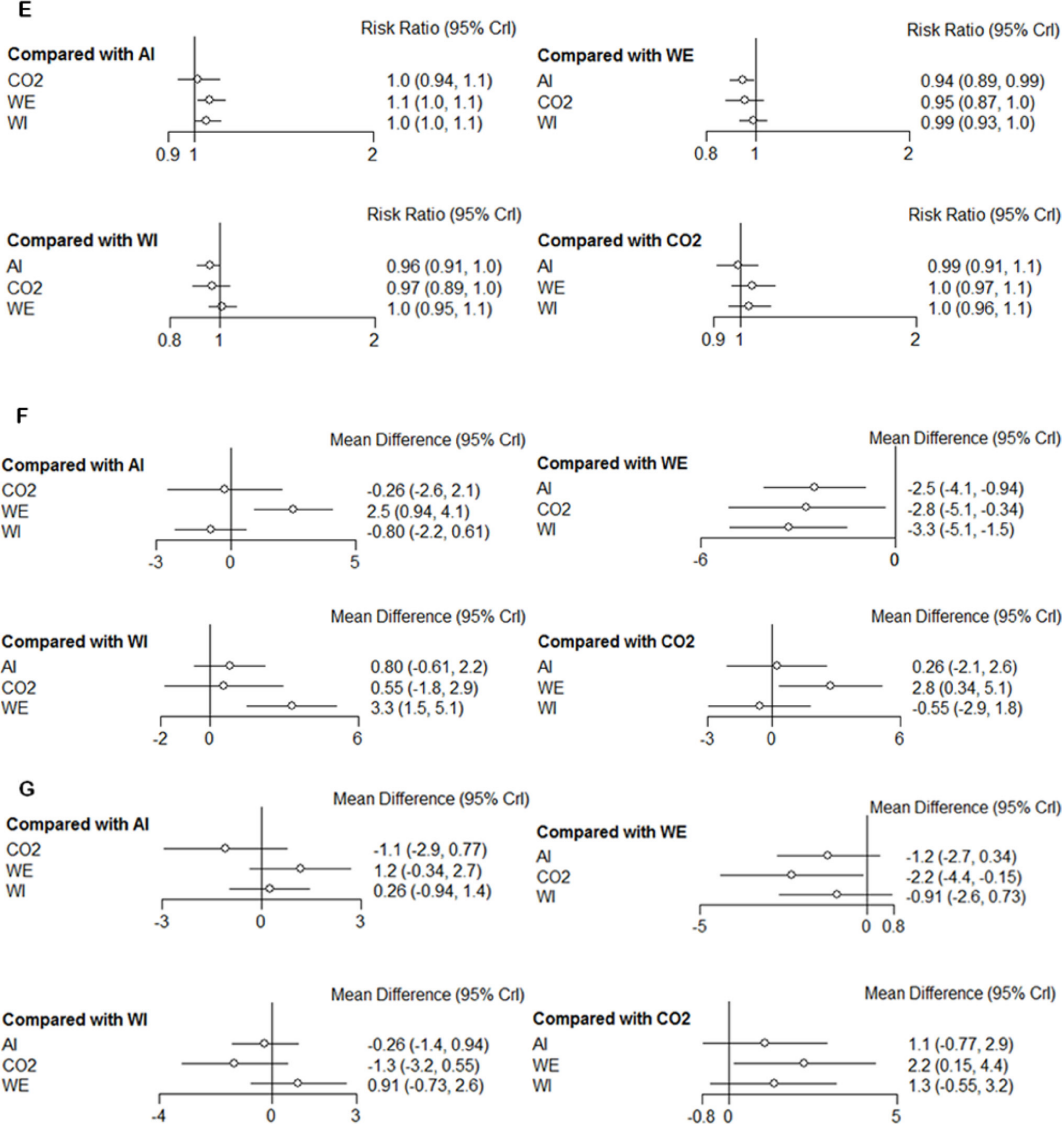
Forest plots of each outcome (**A**) ADR; (**B**) mean pain scores; (**C**) maximum pain scores; (**D**) willingness to repeat; (**E**) caecal intubation rate; (**F**) caecal intubation time; and (**G**) total procedure time.

**Figure 5:**
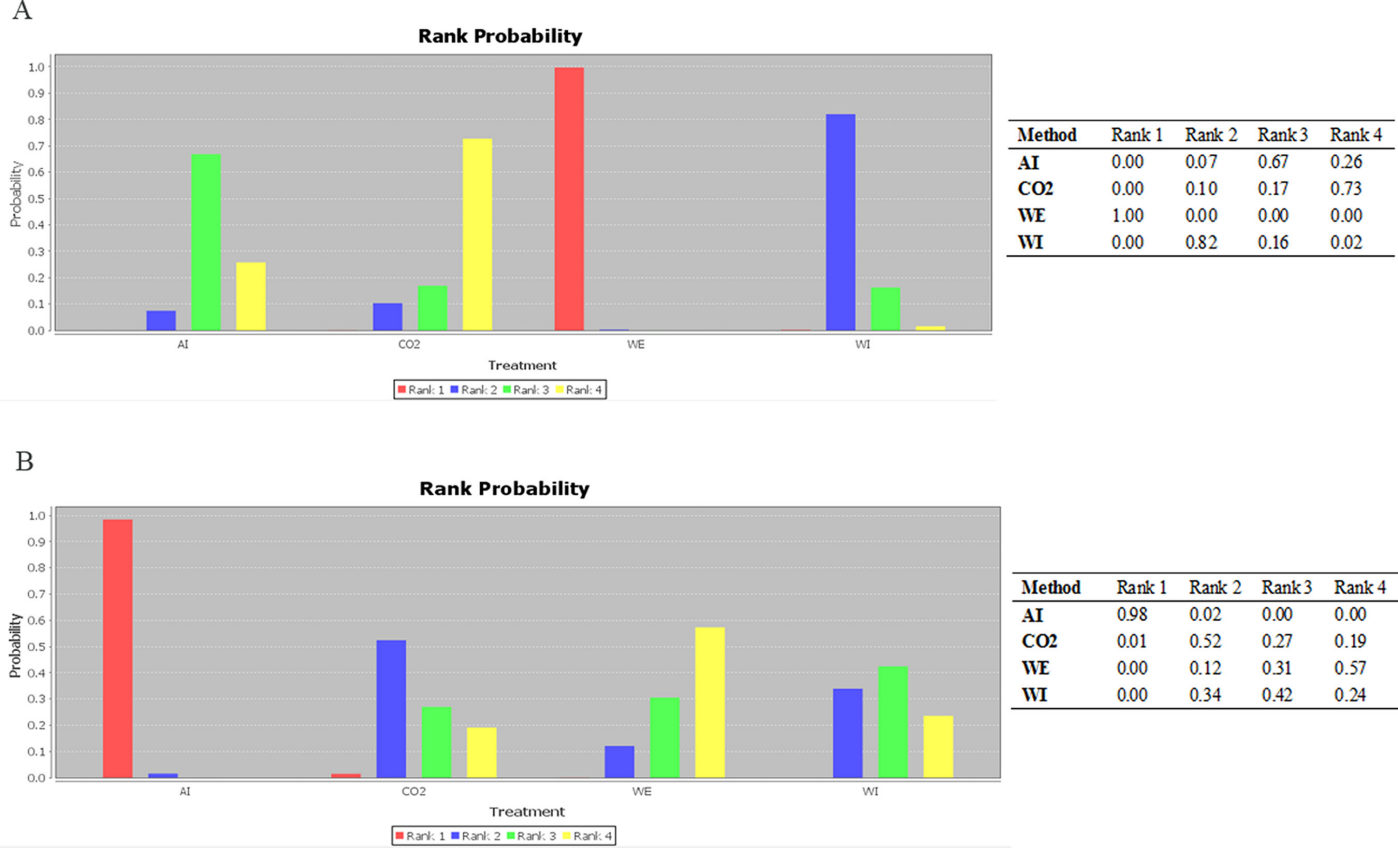

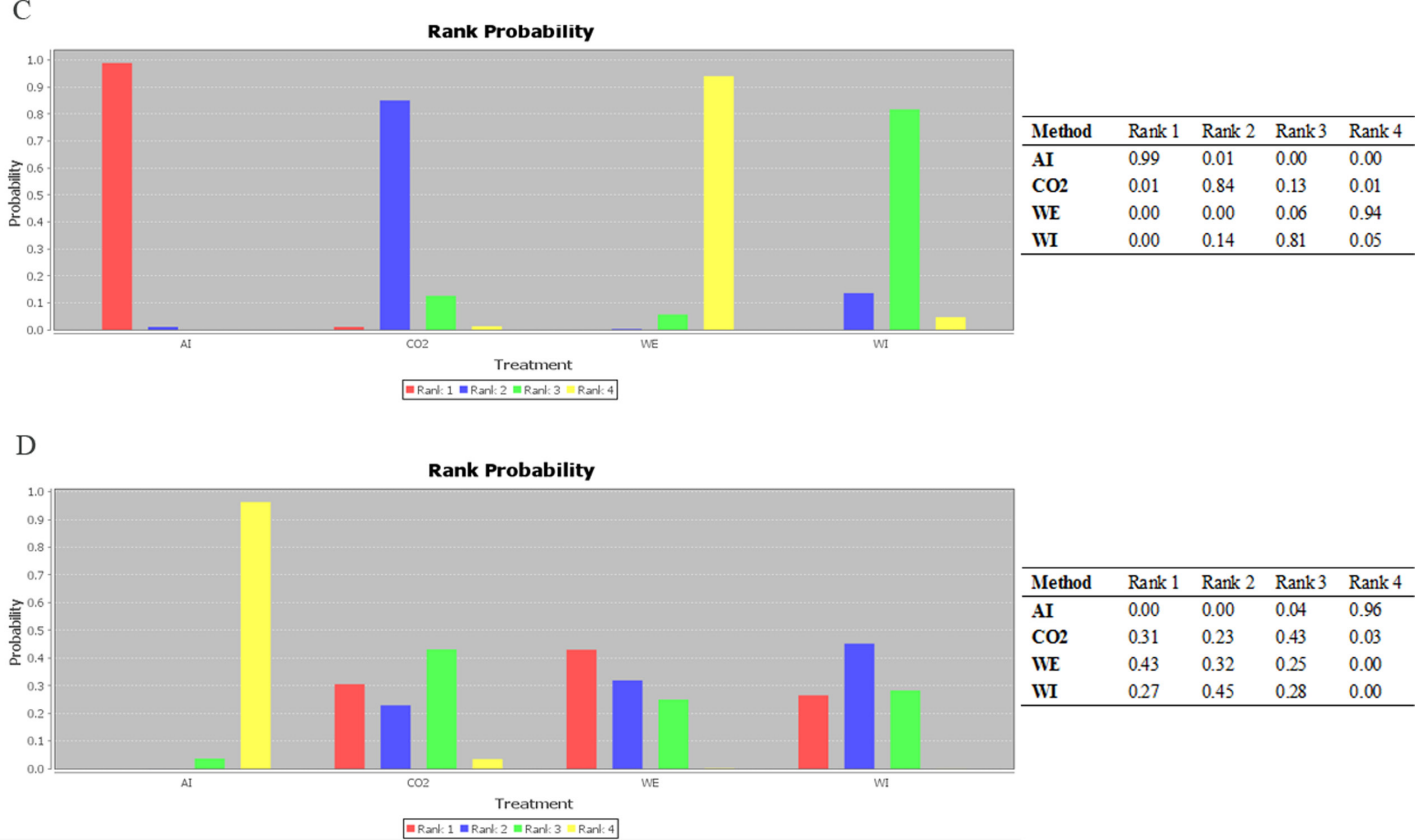

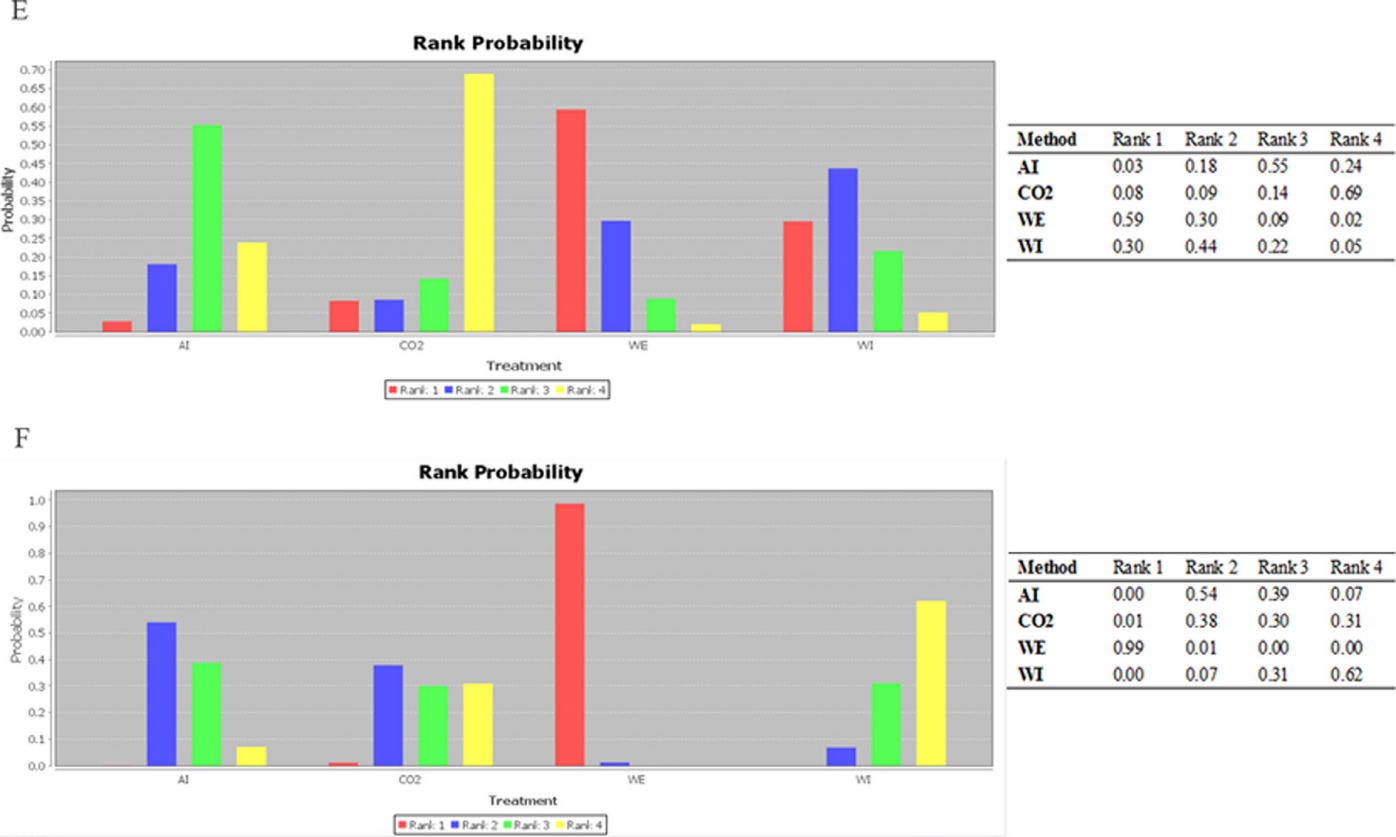

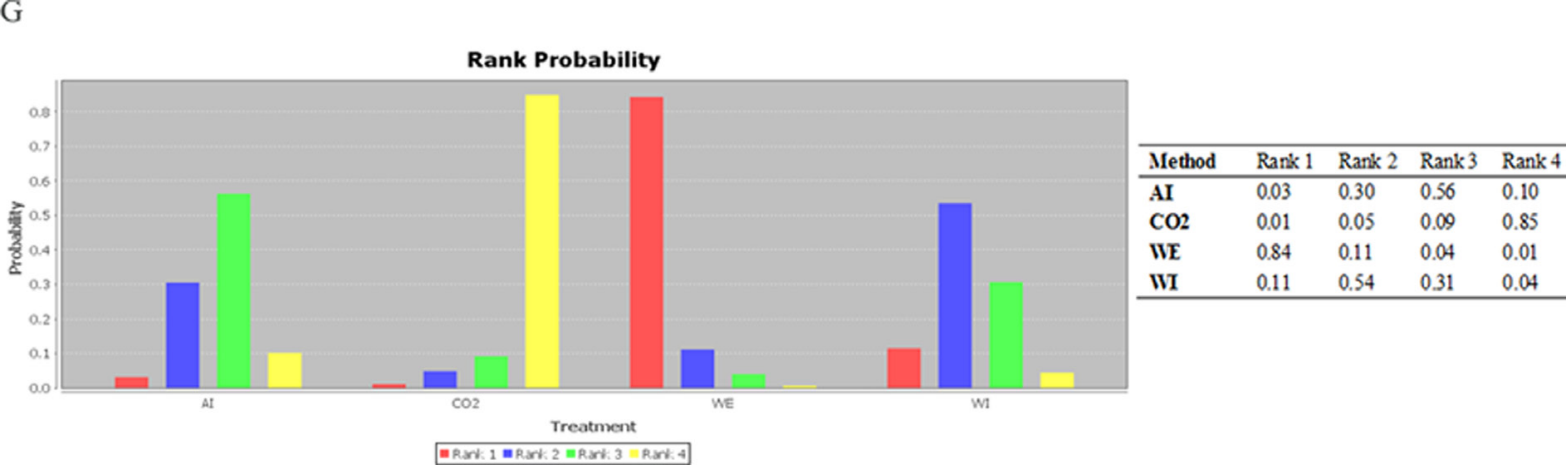
Ranking of method efficacy (**A**) ADR; (**B**) mean pain scores; (**C**) maximum pain scores; (**D**) willingness to repeat; (**E**) caecal intubation rate; (**F**) caecal intubation time; and (**G**) total procedure time.

Figure 4 shows the results of the relative effect between any pair of interventions and each outcome of interest. There was a statistically significant difference in the ADR when WE was compared with WI (RR: 1.2, 95% CrI: 1.1–1.3), AI (RR: 1.3, 95% CrI: 1.1–1.4), and CO_2_ insufflation (RR: 1.2, 95% CrI: 1.1–1.5). In terms of mean and maximum pain scores, willingness to repeat, caecal intubation rate, and total procedure time, there were no significant differences between WI and WE colonoscopy. However, WE required a longer caecal intubation time than WI (MD: 3.3, 95% CrI: 1.5–5.1). Ranking probabilities for all methods are presented in Figure 5. The efficacies of the different methods in adenoma detection were ranked in order from the most to least effective as follows: WE (100%), WI (82%), AI (67%), and CO_2_ (73%).

Node-splitting analysis comparing results between direct and indirect estimates did not produce significant evidence of inconsistency, except when the caecal intubation time of WE was compared with that of AI or CO_2_ (both *P* = 0.04, see Supplementary Table 3). We then compared WE with AI or CO_2_ using an inconsistency model (MD: 2.66, 95% CrI: 1.05–4.19 and MD: 1.73, 95% CrI: –2.38–4.96, respectively).

Egger’s test of ADR for publication bias showed that the risk of having missed or overlooked trials was insignificant: WI vs. WE, *P* = 0.264; WI vs. AI, *P* = 0.331; WE vs. AI, *P* = 0.726. With regard to the remaining comparisons, we were unable to evaluate publication bias due to the small number of included studies.

Subgroup or sensitivity analysis was not conducted because of the limited data available.

## DISCUSSION

Colonoscopies are widely used for prevention and early detection of colorectal cancer. The detection and removal of adenomas, which are major precursor lesions for colorectal cancer, is considered a crucial aspect of cancer prevention [53]. An increase in the ADR has been shown to reduce risks of interval colorectal cancer and colorectal cancer mortality [54], and the ADR has been codified as a key quality indicator for colonoscopy procedures [55, 56]. It has been estimated that every 1% increase in the ADR predicts a 3% decrease in the risk of interval cancer and a 5% decrease in the risk of a fatal interval cancer [57]. Recently published quality guidelines by the European Society of Gastrointestinal Endoscopy recommend that endoscopists achieve an ADR of ≥25% [58], and the American Society of Gastrointestinal Endoscopy recommended an ADR ≥30% in male patients and ≥20% in female patients [55].

The use of water in colonoscopies was first introduced in 1984 as a method for difficult sigmoid circumstances due to diverticulosis [59]. WE was first introduced in 2007 as a modification of WI [60]. The initial purpose of WI was to allow colonoscopists to correctly identify the colonic lumen in order to pass the colonoscope through sigmoid colons that were deformed by diverticulosis and, as a secondary endpoint, to provide a more comfortable procedure for the patients [15]. Thus far, comparative studies have not been able to distinguish the differences in the ADRs between these two colonoscopy approaches. Our network meta-analysis showed that, compared with WI colonoscopy, WE colonoscopy statistically significantly improved the ADR (RR: 1.2, 95% CrI: 1.1–1.3) but did not significantly affect the patients’ pain scores, willingness to repeat rate, caecal intubation rate, or total procedure time. However, WE significantly prolonged the caecal intubation time (MD: 3.3, 95% CrI: 1.5–5.1).

To date, there have been two head-to-head comparisons of WE, WI and AI using the ADR as the primary outcome. Cadoni *et al* [14] conducted an RCT with 408 patients per group, and ADR in the WE group was numerically higher than in the WI group (49.3% vs. 43.4%, *P* = 0.28). However, this difference was not significant. Another study enrolled 651 participants (217 per group) and found that the ADRs in the WE and WI groups were 49.8% and 40.6%, respectively [37]. Although the ADR of the WE group was numerically higher, this difference was also not significant (*P* = 0.064). Therefore, distinguishing the difference between the ADRs of the WE and WI groups may require a larger sample size. In addition, according to a previous study, a sample of 2447 patients per group would be required to show a significant difference in the ADR between WE and WI [14]. Despite the indirect nature of network meta-analysis, we found that WE method of colonoscopy improved the ADR when compared with WI.

The mechanism of the potential superiority of WE over WI is not fully understood. There were some plausible mechanisms of how WE could significantly increase adenoma detection. As shown in Supplementary Table 2 and previous reports [13, 14, 37, 38, 41], WE significantly increased the quality of colon cleanliness to excellent levels both in the entire colon and the ascending colon, and this quality ultimately led to an increase in the ADR. The WE method involves continuous water infusion and suction of residual faeces and air to clear the view, which enables better visualization of adenomas. The turbulence created at the tip of the colonoscope effectively dislodges residual faeces and debris adhered to the mucosa to facilitate removal by aspiration [14]. Inadequate or fair bowel preparation has been proven to be associated with an increased miss rate of adenoma detection [61, 62]. Thus, bowel cleanliness might have contributed to the increased ADR associated with the WE method. The reduced use of suction during the withdrawal step of the WE procedure has been shown to result in few collapses of the lumen or contractions of the colon; moreover, colonoscopists can concentrate on inspecting the colon instead of being distracted by the need to suction residual faeces and water [63]. The longer insertion time required by WE may also play a role in detecting more adenomas during insertion [11]. In addition, Hsieh *et al* [38] found that WE (24.4%) achieved a higher ADR during insertion than WI (15.6%) and AI (14.4%).

Previous studies reported that age ≥50 years, male gender, body mass index ≥25 kg/m^2^, Boston Bowel Preparation Scale (BBPS) score >6, indications of screening and surveillance, WE colonoscopy, and inspection time >8 minutes were significant predictors of increased adenoma detection [13, 37]. We noticed that the included studies showed a wide variation in these variables. Indications such as screening, surveillance, or diagnosis varied among studies, and the recruited patients with a history of colorectal adenoma remained at high risk for colorectal adenoma recurrence [64]. There were three trials [14, 34, 41] only included patients underwent screening colonoscopy, and five articles [26, 33, 36, 44] only included patients underwent diagnosis colonoscopy. Others included patients with indications for screening, surveillance, or diagnosis. This may contribute to potential selection bias. There was a wide variation in gender distribution, which was important because the ADR of men was significantly higher than that of women. Most of patients (≥90%) included in six trials [31–32, 39, 41, 43, 45] were male which could also contribute potential selection bias in our study. However, the data were sparse, and thus, we could not conduct further analysis to control for these confounding factors.

Recent studies revealed that the pain scores associated with WE were considerably lower than those associated with WI [37, 38, 48, 49, 52, 65]. Our study showed that although the differences of the pain scores between WI and WE were not significant, the pain score of WE method was numerically lower. As shown in Figure 5, WE was considered as the least painful method for the ranking probabilities of mean and maximum pain scores (57% and 94% respectively). We needed to be cautious in interpreting the outcome of these pain scores because the pain scores most likely differed between participants who underwent the procedure with or without sedation. As the primary endpoint of the current study was ADR, we included patients regardless of whether they had been sedated. Thus, including patients with full and deep sedation in our study may have lessened the impact of the pain-alleviating effect of WE compared to WI.

In our analysis, we noticed that the pain score from CO_2_ insufflation was second only to that of AI. Previous studies reported that compared with AI or WE colonoscopy, CO_2_ insufflation could significantly reduce post-colonoscopy discomfort and pain but not reduce insertion pain during the colonoscopy [49, 66–70]. CO_2_ is absorbed from the bowel more rapidly than air, which results in a reduction in post-colonoscopy gas volume, and is transported by the blood to the lungs, where it is exhaled [71]. Thus, the purpose of using CO_2_ in the procedure is to prevent or minimize post-colonoscopy colonic distension. A recent study suggested that the combination of WE during insertion and CO_2_ during withdrawal appears to be the optimal choice for decreasing pain during the examination and bloating and other outcomes after the procedure [33].

Our study included the following potential limitations: (1) a limited number of studies directly comparing WE and WI hindered us from conducting subgroup and sensitivity analysis; (2) the inherent indirectness of comparisons in a network meta-analytical approach; (3) in most of the studies, the endoscopists were not blinded to the patient outcomes. Nevertheless, our study is provocative enough to prompt an appropriate response of prospective randomized studies, and consideration given to evaluate the WE method by colonoscopists with low ADR is recommended [72]. Until additional data are available, we believe that WE may be considered superior to WI during colonoscopy.

## SUPPLEMENTARY MATERIALS FIGURE AND TABLES



## Abbreviations

WI: water immersion
WE: water exchange
AI: air insufflation
CO_2_: carbon dioxide
ADR: adenoma detection rate
RCT: randomized controlled trial
RR: risk ratio
MD: mean difference
CrI: credible interval
