# Supplementary material for: Is water exchange superior to water immersion in detecting adenomas during colonoscopies? Results from a Bayesian network meta-analysis
Source: Oncotarget. 2018 Jul 17;9(55):30679–93. doi: 10.18632/oncotarget.25504 (PMC6078142; doi:10.18632/oncotarget.25504)
Supplement: Supplementary file 1 [file oncotarget-09-30679-s001.pdf]

Is water exchange superior to water immersion in detecting adenomas during colonoscopies? Results from a Bayesian network meta-analysis

SUPPLEMENTARY MATERIALS

REFERENCES

1. Hafner S, Zolk K, Radaelli F, Otte J, Rabenstein T, Zolk O. Water infusion versus air insufflation for colonoscopy. Cochrane Database Syst Rev. 2015; CD009863:CD009863. https://doi.org/10.1002/14651858.CD009863.pub2.
2. Lin S, Zhu W, Xiao K, Su P, Liu Y, Chen P, Bai Y. Water intubation method can reduce patients' pain and sedation rate in colonoscopy: a meta-analysis. Dig Endosc. 2013; 25:231–40. https://doi.org/10.1111/den.12018.
3. Jun WU, Bing HU. Comparative effectiveness of water infusion vs air insufflation in colonoscopy: a meta-analysis. Colorectal Dis. 2013; 15:404–09. https://doi.org/10.1111/j.1463-1318.2012.03194.x.
4. Rabenstein T, Radaelli F, Zolk O. Warm water infusion colonoscopy: a review and meta-analysis. Endoscopy. 2012; 44:940–51. https://doi.org/10.1055/s-0032-1310157.
5. Leung FW, Amato A, Ell C, Friedland S, Harker JO, Hsieh YH, Leung JW, Mann SK, Paggi S, Pohl J, Radaelli F, Ramirez FC, Siao-Salera R, Terruzzi V. Water-aided colonoscopy: a systematic review. Gastrointest Endosc. 2012; 76:657–66. https://doi.org/10.1016/j.gie.2012.04.467.
6. Leung F, Harker J, Leung J, Siao-Salera R, Mann S, Ramirez F, Friedland S, Amato A, Radaelli F, Paggi S, Terruzzi V, Hsieh Y. Removal of infused water predominantly during insertion (water exchange) is consistently associated with an increase in adenoma detection rate - review of data in randomized controlled trials (RCTs) of water-related methods. J Interv Gastroenterol. 2011; 1:121–26. https://doi.org/10.4161/jig.1.3.18517.
7. Cadoni S, Falt P, Rondonotti E, Radaelli F, Fojtik P, Gallittu P, Liggi M, Amato A, Paggi S, Smajstrla V, Urban O, Erriu M, Koo M, Leung FW. Water exchange for screening colonoscopy increases adenoma detection rate: a multicenter, double-blinded, randomized controlled trial. Endoscopy. 2017; 49:456–67. https://doi.org/10.1055/s-0043-101229.
8. Jia H, Pan Y, Guo X, Zhao L, Wang X, Zhang L, Dong T, Luo H, Ge Z, Liu J, Hao J, Yao P, Zhang Y, et al. Water Exchange Method Significantly Improves Adenoma Detection Rate: A Multicenter, Randomized Controlled Trial. Am J Gastroenterol. 2017; 112:568–76. https://doi.org/10.1038/ajg.2016.501.
9. Cadoni S, Falt P, Gallittu P, Liggi M, Smajstrla V, Leung FW. Impact of carbon dioxide insufflation and water exchange on postcolonoscopy outcomes in patients receiving on-demand sedation: a randomized controlled trial. Gastrointest Endosc. 2017; 85:210–218.e1. https://doi.org/10.1016/j.gie.2016.05.021.
10. Hsieh YH, Tseng CW, Hu CT, Koo M, Leung FW. Prospective multicenter randomized controlled trial comparing adenoma detection rate in colonoscopy using water exchange, water immersion, and air insufflation. Gastrointest Endosc. 2017; 86:192–201. https://doi.org/10.1016/j.gie.2016.12.005.
11. Arai M, Okimoto K, Ishigami H, Taida T, Oyamada A, Minemura S, Saito K, Tsuboi M, Maruoka D, Matsumura T, Nakagawa T, Katsuno T, Mitsuhashi K, et al. A randomized controlled trial comparing water exchange and air insufflation during colonoscopy without sedation. Int J Colorectal Dis. 2016; 31:1217–23. https://doi.org/10.1007/s00384-016-2580-z.
12. Xu X, Zhu H, Chen D, Fan L, Lu T, Shen Q, Chen C, Deng D. Carbon dioxide insufflation or warm-water infusion for unsedated colonoscopy: A randomized controlled trial in patients with chronic constipation in China. Saudi J Gastroenterol. 2016; 22:18–24. https://doi.org/10.4103/1319-3767.173754.
13. Falt P, Šmajstrla V, Fojtík P, Urban O, Hill M. Water-Aided Colonoscopy in Inflammatory Bowel Disease Patients-A Randomised, Single-Centre Trial. J Crohn's Colitis. 2015; 9:720–24. https://doi.org/10.1093/ecco-jcc/jjv093.
14. Cadoni S, Sanna S, Gallittu P, Argiolas M, Fanari V, Porcedda ML, Erriu M, Leung FW. A randomized, controlled trial comparing real-time insertion pain during colonoscopy confirmed water exchange to be superior to water immersion in enhancing patient comfort. Gastrointest Endosc. 2015; 81:557–66. https://doi.org/10.1016/j.gie.2014.07.029.

15. Cadoni S, Falt P, Gallittu P, Liggi M, Mura D, Smajstrla V, Erriu M, Leung FW. Water Exchange Is the Least Painful Colonoscope Insertion Technique and Increases Completion of Unsedated Colonoscopy. Clin Gastroenterol Hepatol. 2015; 13:1972–80.e1-3. https://doi.org/10.1016/j.cgh.2015.04.178.
16. Wang X, Luo H, Xiang Y, Leung FW, Wang L, Zhang L, Liu Z, Wu K, Fan D, Pan Y, Guo X. Left-colon water exchange preserves the benefits of whole colon water exchange at reduced cecal intubation time conferring significant advantage in diagnostic colonoscopy - a prospective, randomized controlled trial. Scand J Gastroenterol. 2015; 50:916–23. https://doi.org/10.3109/00365521.2015.1010569.
17. Miroslav V, Klemen M. Warm water immersion vs. standard air insufflation for colonoscopy: comparison of two techniques. Hepatogastroenterology. 2014; 61:2209–11.
18. Hsieh YH, Koo M, Leung FW. A patient-blinded randomized, controlled trial comparing air insufflation, water immersion, and water exchange during minimally sedated colonoscopy. Am J Gastroenterol. 2014; 109:1390–400. https://doi.org/10.1038/ajg.2014.126.
19. Cadoni S, Gallittu P, Sanna S, Fanari V, Porcedda ML, Erriu M, Leung FW. A two-center randomized controlled trial of water-aided colonoscopy versus air insufflation colonoscopy. Endoscopy. 2014; 46:212–18. https://doi.org/10.1055/s-0033-1353604.
20. Leung JW, Mann S, Siao-Salera R, Canete W, Prather D, Leung FW. The established and time-tested water exchange method in scheduled unsedated colonoscopy significantly enhanced patient-centered outcomes without prolonging procedural times– A randomized controlled trial. J Interv Gastroenterol. 2013; 3:7.
21. Amato A, Radaelli F, Paggi S, Baccarin A, Spinzi G, Terruzzi V. Carbon dioxide insufflation or warm-water infusion versus standard air insufflation for unsedated colonoscopy: a randomized controlled trial. Dis Colon Rectum. 2013; 56:511–18. https://doi.org/10.1097/DCR.0b013e318279addd.
22. Luo H, Zhang L, Liu X, Leung FW, Liu Z, Wang X, Xue L, Wu K, Fan D, Pan Y, Guo X. Water exchange enhanced cecal intubation in potentially difficult colonoscopy. Unsedated patients with prior abdominal or pelvic surgery: a prospective, randomized, controlled trial. Gastrointest Endosc. 2013; 77:767–73. https://doi.org/10.1016/j.gie.2012.12.007.
23. Bayupurnama P, Neneng R, Fahmi I, Catharina T, Siti N. W LF. The water method colonoscopy in routine unsedated colonoscopy examinations: a randomized controlled trial in diagnostic cases in Indonesian patients. J Interv Gastroenterol. 2013; 3:12. https://doi.org/10.7178/jig.101.
24. Hsieh YH, Leung FW. A randomized, controlled trial comparing air insufflation, water immersion and water exchange during minimally sedated colonoscopy - an interim report. J Interv Gastroenterol. 2013; 3:96–99.
25. Portocarrero DJ, Che K, Olafsson S, Walter MH, Jackson CS, Leung FW, Malamud A. A pilot study to assess feasibility of the water method to aid colonoscope insertion in community settings in the United States. J Interv Gastroenterol. 2012; 2:20–22. https://doi.org/10.4161/jig.20130.
26. Falt P, Liberda M, Smajstrla V, Kliment M, Bártková A, Tvrdík J, Fojtík P, Urban O. Combination of water immersion and carbon dioxide insufflation for minimal sedation colonoscopy: a prospective, randomized, single-center trial. Eur J Gastroenterol Hepatol. 2012; 24:971–77. https://doi.org/10.1097/MEG.0b013e3283543f16.
27. Hsieh YH, Tseng KC, Hsieh JJ, Tseng CW, Hung TH, Leung FW. Feasibility of colonoscopy with water infusion in minimally sedated patients in an Asian Community Setting. J Interv Gastroenterol. 2011; 1:185–90. https://doi.org/10.4161/jig.1.4.19961.
28. Ramirez FC, Leung FW. A head-to-head comparison of the water vs. air method in patients undergoing screening colonoscopy. J Interv Gastroenterol. 2011; 1:130–35. https://doi.org/10.4161/jig.1.3.18512.
29. Pohl J, Messer I, Behrens A, Kaiser G, Mayer G, Ell C. Water infusion for cecal intubation increases patient tolerance, but does not improve intubation of unsedated colonoscopies. Clin Gastroenterol Hepatol. 2011; 9:1039–43.e1. https://doi.org/10.1016/j.cgh.2011.06.031.
30. Leung J, Mann S, Siao-Salera R, Ransibrahmanakul K, Lim B, Canete W, Samson L, Gutierrez R, Leung FW. A randomized, controlled trial to confirm the beneficial effects of the water method on U.S. veterans undergoing colonoscopy with the option of on-demand sedation. Gastrointest Endosc. 2011; 73:103–10. https://doi.org/10.1016/j.gie.2010.09.020.
31. Leung FW, Harker JO, Jackson G, Okamoto KE, Behbahani OM, Jamgotchian NJ, Aharonian HS, Guth PH, Mann SK, Leung JW. A proof-of-principle, prospective, randomized, controlled trial demonstrating improved outcomes in scheduled unsedated colonoscopy by the water method. Gastrointest Endosc. 2010; 72:693–700. https://doi.org/10.1016/j.gie.2010.05.020.
32. Leung CW, Kaltenbach T, Soetikno R, Wu KK, Leung FW, Friedland S. Water immersion versus standard colonoscopy insertion technique: randomized trial shows promise for minimal sedation. Endoscopy. 2010; 42:557–63. https://doi.org/10.1055/s-0029-1244231.
33. Radaelli F, Paggi S, Amato A, Terruzzi V. Warm water infusion versus air insufflation for unsedated colonoscopy: a randomized, controlled trial. Gastrointest Endosc. 2010; 72:701–09. https://doi.org/10.1016/j.gie.2010.06.025.
34. Ransibrahmanakul K, Leung JW, Mann SK, Siao-Salera R, Lim BS, Hasyagar C, Yen D, Nastaskin I, Leung FW. Comparative Effectiveness of Water vs. Air Methods in Minimal Sedation Colonoscopy Performed by Supervised Trainees in the US - Randomized Controlled Trial. Am J Chin Med. 2010; 7:113–18.
35. Leung JW, Mann SK, Siao-Salera R, Ransibrahmanakul K, Lim B, Cabrera H, Canete W, Barredo P, Gutierrez R, Leung FW. A randomized, controlled comparison of warm water infusion in lieu of air insufflation versus air insufflation for aiding colonoscopy insertion in sedated patients undergoing colorectal cancer screening and surveillance. Gastrointest Endosc. 2009; 70:505–10. https://doi.org/10.1016/j.gie.2008.12.253.

Supplementary Table 1: Previous meta-analyses comparing water infusion against air insufflation in adenoma detecting during colonoscopy

Author	Year	Comparison	Risk ratio	*P* value
Hafner S *et al* [1]	2015	Water infusion vs. air insufflation	1.16 (1.04–1.30)	0.007
Lin S *et al* [2]	2013	Water infusion vs. air insufflation	0.91 (0.68–1.22)[1]	0.168
Jun W *et al* [3]	2013	Water infusion vs. air insufflation	1.01 (0.79–1.29)	0.96
Rabenstein T *et al* [4]	2012	Water infusion vs. air insufflation	0.95 (0.81–1.10)	0.49
Leung F *et al* [5]	2012	Water infusion vs. air insufflation	-	NS
Leung F *et al* [6]	2011	Water immersion vs. air insufflation	-7%[2]	0.023
		Water exchange vs. air insufflation	8%[2]	0.019

NS, not significant.

[1]odds ratio.

[2]difference in adenoma detection rate.

Supplementary Table 2: Outcome measures of colonoscopy

Author	Year	Patients, *n*				Quality of bowel preparation*				Adenoma detection rate, *n*				Pain Score				Willingness to repeat, *n*				Caecal intubation rate, *n*				Caecal intubation time (min)				Total procedure time (min)			
		WI	WE	AI	CO_2	WI	WE	AI	CO_2	WI	WE	AI	CO_2	WI	WE	AI	CO_2	WI	WE	AI	CO_2	WI	WE	AI	CO_2	WI	WE	AI	CO_2	WI	WE	AI	CO_2
Cadoni S [7]	2017	408	408	408	–	8.0 ± 2.2	9.0 ± 1.5	8.0 ± 2.2	–	177	201	165	–	–	–	–	–	390	389	386	–	377	387	386	–	8 ± 2.1	9 ± 2	8 ± 2.2	–	22 ± 2.9	21 ± 3.1	20.5 ± 3.1	
Jia H [8]	2017	–	1653	1650	–	–	7.3 ± 1.6	7.0 ± 2.3	–	–	303	221	–	–	2.9 ± 1.3	3.6 ± 1.2	–	–	1464	1412	–	–	1632	1626	–	–	7.4 ± 1.8	4.9 ± 1.5	–	–	–	–	–
Cadoni S [9]	2017	–	80	–	81	–	7.6 ± 1.7	–	6.8 ± 1.8	–	–	–	–	–	3.3 ± 0.3	–	4.7 ± 0.3	–	–	–	–	–	76	–	77	–	9.4 ± 3.9	–	9.8 ± 6.1	–	20.1 ± 6.9	–	20.1 ± 6.9
Hsieh YH [10]	2017	217	217	217	–	6.6 ± 1.2	7.1 ± 1.3	6.2 ± 1.1	–	88	108	82	–	2.4 ± 2.7	1.7 ± 2.6	3.9 ± 3.1	–	–	–	–	–	191	208	197	–	7 ± 4.7	14 ± 6.3	7.5 ± 5	–	19.3 ± 8.7	25.1 ± 13.4	18.9 ± 9.2	–
Arai M [11]	2016	–	206	–	197	–	–	–	–	–	139	–	114	–	4.3 ± 2.1	–	4.8 ± 2.1	–	–	–	–	–	–	–	–	–	8.3 ± 4.9	–	9.1 ± 7.4	–	–	–	–
Xu X [12]	2016	97	–	94	96	–	–	–	–	–	–	–	–	2.7 ± 1.9	–	5.7 ± 2.5	2.9 ± 2.1	91	–	58	90	97	–	94	96	6.9 ± 1.3	–	10.6 ± 2.5	7.2 ± 1.4	14.9 ± 1.7	–	18.2 ± 3.1	15.1 ± 1.8
Falt P [13]	2015	–	46	46	–	–	82.6%	67.4%	–	–	–	–	–	–	3.8 ± 2.4	5.4 ± 1.9	–	–	–	–	–	–	39	37	–	–	7.6 ± 2.7	7.4 ± 3.4	–	–	15.7 ± 4.9	14.8 ± 5	–
Cadoni S [14]	2015	197	186	193	–	7.1 ± 1.9	7.8 ± 1.6	7.1 ± 1.9	–	–	–	–	–	3.5 ± 3.2	2.5 ± 2.4	4.1 ± 2.8	–	188	177	148	–	190	181	185	–	10 ± 5	12 ± 5.7	12 ± 7.7	–	22 ± 13.1	23 ± 9.3	23 ± 11.2	–
Cadoni S [15]	2015	103	105	103	105	7.3 ± 1.4	8.0 ± 1.3	7.2 ± 1.7	7.0 ± 1.6	–	–	–	–	4 ± 2.7	3.1 ± 2.4	5.2 ± 3	4.9 ± 3	93	103	89	97	102	105	101	104	9.8 ± 6.2	10.9 ± 6	9.7 ± 6.9	9.3 ± 5.6	20.8 ± 8.9	22.9 ± 9.9	23.2 ± 12.7	20.5 ± 8.4
Wang X [16]	2015	–	99	98	–	–	8.4 ± 1.0	7.6 ± 1.2	–	–	–	–	–	–	1.1 ± 1.1	2.9 ± 2	–	–	97	84	–	–	97	86	–	–	8.2 ± 10.8	7 ± 14	–	–	–	–	–
Miroslav V [17]	2014	57	–	65	–	–	–	–	–	–	–	–	–	4.1 ± 2.6		5.1 ± 2.6	–	55	–	63	–	–	–	–	–	–	–	–	–	–	–	–	
Hsieh YH [18]	2014	90	90	90	–	6.7 ± 1.2	6.8 ± 1.3	6.1 ± 1.1	–	41	51	39	–	2.5 ± 2.7	1.4 ± 2.4	3.6 ± 3	–	90	90	89	–	83	82	71	–	6.5 ± 3.7	17.4 ± 5.9	9.6 ± 7.9	–	–	–	–	–
Cadoni S [19]	2014	–	338	334	–	–	–	–	–	–	87	64	–	–	1.3 ± 1.9	2.3 ± 2.3	–	–	–	–	–	–	326	330	–	–	11 ± 7.7	9 ± 5.9	–	–	–	–	–
Leung J [20]	2013	–	50	50	–	–	–	–	–	–	27	24	–	–	3 ± 2.8	5 ± 3	–	–	38	24	–	–	48	24	–	–	13 ± 6.7	12 ± 7	–	–	29 ± 12	28 ± 12	–
Amato A [21]	2013	113	–	113	115	93.8%	–	94.6%	93.9%	44	–	42	35	2.8 ± 1.6	–	4.6 ± 1.7	3 ± 1.7	99	–	88	102	110	–	112	110	9 ± 2.1	–	7 ± 1.9	5 ± 2.1	17 ± 2.6	–	14 ± 2.9	13 ± 2.7
Luo H [22]	2013	–	55	55	–	–	8.1 ± 1.2	7.2 ± 1.6	–	–	–	–	–	–	2.1 ± 1.8	4.6 ± 1.7	–	–	50	40	–	–	51	42	–	–	11.9 ± 4.3	11.5 ± 6.6	–	–	18.3 ± 4	17.7 ± 5.7	–
Bayupurnama P [23]	2013	53	–	57	–	–	–	–	–	–	–	–	–	4.1 ± 2.6	–	6.4 ± 2.4	–	41	–	32	–	49	–	51	–	11.9 ± 5.5	–	12.9 ± 7.1	–	18 ± 6.3	–	18.2 ± 8	–
Hsieh YH [24]	2013	64	68	68	–	6.6 ± 1.3	6.7 ± 1.4	6.1 ± 1.1	–	–	–	–	–	2.4 ± 2.6	1.5 ± 2.4	3.8 ± 3	–	64	68	67	–	60	60	57	–	6.6 ± 3.6	17.5 ± 6.4	8.9 ± 7.3	–	–	–	–	–
Portocarrero DJ [25]	2012	11	–	12	–	–	–	–	–	6	–	2	–	3.6 ± 2	–	3.6 ± 1.6	–	11	–	11	–	11	–	12	–	–	–	–	–	14 ± 3	–	16 ± 7	–
Falt P [26]	2012	100	–	101	102	78.0%	–	74.3%	83.3%	33	–	22	27	1.6	–	2.3	2	–	–	–	–	97	–	85	84	8.8 ± 4.8	–	7.8 ± 4.5	8.5 ± 4.5	20 ± 6.5	–	17.6 ± 6	17.9 ± 5.9
Hsieh YH [27]	2011	51	–	51	–	88.2%	–	90.2%	–	15	–	13	–	3.3 ± 2.4	–	4.4 ± 2.6	–	49	–	48	–	50	–	50	–	5.6 ± 3.4	–	4.6 ± 2.6	–	15.3 ± 5.9	–	13.1 ± 5.4	–
Ramirez FC [28]	2011	–	177	191	–	–	84.2%	78.5%	–	–	101	88	–	–	–	–	–	–	–	–	–	–	163	191	–	–	6.9 ± 0.3	5.3 ± 0.3	–	–	19.9 ± 0.5	18.9 ± 0.6	–
Pohl J [29]	2011	58	–	58	–	–	–	–	–	19	–	15	–	2.8 ± 1.9	–	4.2 ± 2.3	–	42	–	39	–	48	–	56	–	8.1 ± 3	–	6.2 ± 3.4	–	19.2 ± 7.2	–	15.7 ± 4.8	–
Leung J [30]	2011	50	–	50	–	–	–	–	–	20	–	18	–	2.3 ± 1.7	–	4.9 ± 2	–	45	–	47	–	50	–	50	–	13.1 ± 8.1	–	11 ± 5.6	–	28.1 ± 12.6	–	24.4 ± 8.6	–
Leung FW [31]	2010	42	–	40	–	–	–	–	–	15	–	9	–	3.6 ± 2.1	–	5.5 ± 3	–	39	–	31	–	41	–	31	–	34 ± 13	–	37 ± 16	–	56 ± 13	–	56 ± 17	–
Leung CW [32]	2010	112	–	114	–	80.4%	–	80.0%	–	47	–	45	–	4.1 ± 2.7	–	5.3 ± 2.7	–	–	–	–	–	112	–	114	–	10.2 ± 7.9	–	15.3 ± 13.1	–	25.1 ± 14.3	–	30.3 ± 14.5	–
Radaelli F [33]	2010	116	–	114	–	94.0%	–	94.7%	–	29	–	46	–	2.8 ± 1.5	–	3.9 ± 1.7	–	105	–	93	–	109	–	109	–	7 ± 2.1	–	5 ± 1.7	–	15 ± 2.4	–	14 ± 2.3	–
Ransi-brahmanakul K [34]	2010	31	–	31	–	–	–	–	–	9	–	10	–	3.1 ± 2.9	–	4.8 ± 3.3	–	29	–	25	–	31	–	31	–	11 ± 7.3	–	10 ± 5.5	–	21 ± 8.5	–	24 ± 11.6	–
Leung JW [35]	2009	28	–	28	–	–	–	–	–	–	–	–	–	1.3 ± 0.3	–	4.1 ± 0.6	–	27	–	27	–	28	–	28	–	8.8 ± 3.8	–	11 ± 8	–	21 ± 7.2	–	21 ± 8.6	

WI, water immersion; WE, water exchange; AI, air insufflation; CO_2, carbon dioxide.
*expressed as Boston Bowel Preparation Scale or percentage of patients with good-excellent bowel preparation.

Supplementary Table 3: Node-splitting test

	P-value						
**Groups**	**ADR**	**Mean pain score**	**Maximum pain score**	**Williness to repeat**	**Caecal intubation rate**	**Caecal intubation time**	**Total procedure time**
AI vs. CO_2	0.57	0.70	0.38	-	0.66	0.07	0.28
AI vs. WE	0.54	0.76	0.36	-	0.69	0.04	0.27
CO_2 vs. WE	0.57	0.81	0.26	0.30	0.91	0.04	0.42
CO_2 vs. WI	0.30	0.85	0.77	-	0.59	0.18	0.87
WE vs. WI	0.90	0.49	0.12	0.77	0.91	0.06	0.44

ADR, adenoma detection rate; WI, water immersion; WE, water exchange; AI, air insufflation; CO_2, carbon dioxide.

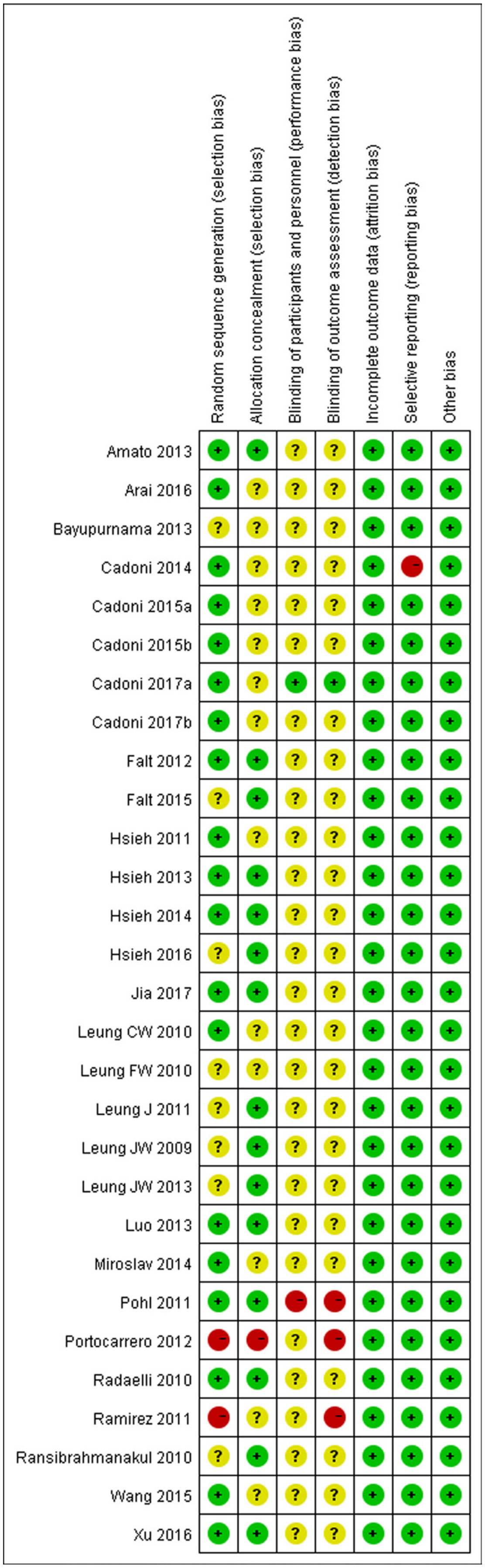

Supplementary Figure 1: Risk of bias summary.
